# Mental health care use and quality among Medicaid adults with serious mental illness receiving care at Federally Qualified Health Centers vs. other settings

**DOI:** 10.1186/s12913-024-11308-1

**Published:** 2024-07-17

**Authors:** Catherine Myong, Zhiyou Yang, Caroline Behr, Vicki Fung

**Affiliations:** 1 Health Policy Research Center, Mongan Institute, Massachusetts General Hospital, Boston, MA United States of America; 2 Department of Psychiatry, Columbia University Irving Medical Center, New York, NY United States of America; 3 Department of Medicine, Brigham and Women’s Hospital, Boston, MA United States of America; 4 Department of Medicine, Harvard Medical School, Boston, MA United States of America

**Keywords:** FQHCs, Community health centers, Mental health care, Primary care, Serious mental illness

## Abstract

**Background:**

Federally Qualified Health Centers (FQHCs) are a critical source of care for medically underserved populations and often serve as medical homes for individuals with serious mental illness (SMI). Many FQHCs provide mental health services and could facilitate access to mental health treatment within and outside of FQHCs. This study compared mental health care utilization and acute care events for adult Medicaid enrollees with SMI who receive care at Federally Qualified Health Centers (FQHCs) vs. other settings.

**Methods:**

This study used the 2015–2016 Massachusetts All-Payer Claims Database to examine outpatient mental health care and acute care events for 32,330 Medicaid adults, ages 18–64 and with major depressive, bipolar, or schizophrenia spectrum disorders (SSD), who resided in FQHC service areas and received care from FQHCs vs. other settings in 2015. Multivariable linear regressions assessed associations between receiving care at FQHCs and outpatient mental health visits, psychotropic medication fills, and acute care events in 2016.

**Results:**

There were 8,887 (27.5%) adults in the study population (*N* = 32,330) who had at least one FQHC visit in 2015. Those who received care at FQHCs were more likely to have outpatient mental health visits (73.3% vs. 71.2%) and psychotropic medication fills (73.2% vs. 69.0%, both *p* < .05), including antidepressants among those with depressive disorders and antipsychotics among those with SSD. They were more likely to have ED visits (74.0% vs. 68.7%), but less likely to be hospitalized (27.8% vs. 31.9%, both *p* < .05). However, there was no significant difference in the likelihood of having an acute psychiatric hospitalization (9.5% vs. 9.8%, *p* = .35).

**Conclusions:**

Among Medicaid enrollees with SMIs who had access to care at FQHCs, those receiving care at FQHCs were more likely to have outpatient mental health visits and psychotropic medication fills, with lower rates of hospitalization, suggesting potentially improved quality of outpatient care. Higher ED visit rates among those receiving care at FQHCs warrant additional investigation.

**Supplementary Information:**

The online version contains supplementary material available at 10.1186/s12913-024-11308-1.

## Background

Individuals with serious mental illness (SMI) have complex physical and mental health care needs. Evidence suggests those with SMI face a pre-mature mortality gap of 8 to 30 years compared with those without SMI, largely owing to medical comorbidities such as metabolic syndrome and cardiovascular disease, underscoring the importance of high quality primary care for this population [1–3]. Individuals with vs. without SMI are also more likely to have incomes below the federal poverty level, more likely to be uninsured or have Medicaid insurance, and are at increased vulnerability to social determinants of health [4–9]. Federally Qualified Health Centers (FQHCs) are a potential care setting for people with SMI to receive high-quality, accessible care [10, 11], but there is limited information on care patterns for individuals with SMI who receive care from FQHCs compared with other settings.

FQHCs provide care to underserved areas and populations, and the majority of patients at FQHCs have incomes at or below 200% of the federal poverty level [12]. FQHCs are also required to offer comprehensive primary care with enabling services, such as transportation, language interpretation, care coordination and other non-clinical services that aim to increase access to health care [10]. In addition, almost 90% of FQHCs provide on-site mental health services per a 2019 national survey, [13] although the types of services offered and availability of providers vary and may vary across delivery sites for a given health center [13, 14]. 

From 2010 to 2015, the volume of mental health visits and patients at FQHCs increased by 8–14% annually, at a rate greater than the general increase in FQHC patient volumes [15]. There has been growing recognition of the need to support mental health care capacity at FQHCs, particularly as coverage for mental health services has expanded with the passage of the Mental Health Parity and Addiction Equity Act in 2008 and the Affordable Care Act in 2010 [11, 16]. Many FQHCs have implemented components of behavioral health integration to better coordinate behavioral and primary health care and increase delivery of mental health care both on-site or via referral [3, 17, 18]. As key primary care providers in underserved communities, FQHCs could also play a critical role in connecting individuals with SMI to community-based treatments designed to address complex psychiatric needs [11]. 

There is little existing evidence, however, on differences in utilization patterns and quality of care for individuals with SMI who receive care at FQHCs versus other care settings. A study of the North Carolina Medicaid population found no difference in emergency department (ED) use and medication adherence for adults with SMI in FQHC medical homes compared to other medical homes [10, 19]. In this study, we used the Massachusetts All-Payer Claims Dataset (APCD) to examine differences in outpatient mental health care, including mental health visits and psychotropic medication use, and acute care events, including ED visits and hospitalizations, among adult Medicaid enrollees with SMI who reside in FQHC service areas and receive care at FQHCs vs. other outpatient settings.

## Methods

### Data source and population

This study used enrollment, medical and pharmacy claims data for adults of ages 18–64 from the Massachusetts All Payer Claim Database (APCD) Release 6.0, which mainly includes individuals with Medicare Advantage, Medicaid, and commercial insurance. We excluded individuals aged 65 + and beneficiaries dually eligible for Medicaid and Medicare benefits. We obtained FQHC patient counts by ZIP code, which were used to determine FQHC service areas, from the 2015 Uniform Data System (UDS) provided by the Health Resources and Services Administration (HRSA). Lastly, ZIP-code level measures of race/ethnicity, household income, and educational attainment were obtained from the American Community Survey 5-Year Data for 2011–2015. We examined outcomes in 2016 and defined baseline characteristics using prior years of data – e.g., FQHC use in 2015.

The analysis included 38 FQHCs in Massachusetts in 2015–2016 that received Health Center funding from HRSA. To reduce potential confounding associated with differences in access to care related to insurance and neighborhood, we limited our study population to individuals who had continuous Medicaid enrollment in 2015 and were living in ZIP codes included in FQHC service areas [20]. We conducted a sensitivity analysis further limiting the study population to those with continuous Medicaid enrollment in both 2015 and 2016 with consistent results (Supplement File 1). We applied a previously validated empirical definition of FQHC service areas that uses the ZIP code locations of FQHC patients available in the UDS; [21] using 2015 UDS data, we identified 169 ZIP codes in Massachusetts that were included in FQHC service areas. This definition of FQHC service areas encompassed 72% of adults with Medicaid insurance in the APCD.

We identified individuals with diagnoses of major depressive, bipolar, or schizophrenia spectrum disorders (SSD) using International Classification of Diseases Ninth Revision (ICD-9) and ICD-10 diagnoses codes used by the Centers for Medicare and Medicaid Services Chronic Condition Data Warehouse. Individuals were required to have at least 2 outpatient or 1 inpatient diagnoses of a given condition over a two year period (2014–2015). We used a hierarchical definition previously used in studies of individuals with SMI to define mutually exclusive groups: i.e., those with diagnoses of SSD plus bipolar and/or major depressive disorders were classified as having SSD; those with bipolar disorder diagnoses and major depressive disorder were classified as having bipolar disorder [22, 23]. Those excluded due to missing data included 12,242 out of 8.2 million (0.15%) individuals in the APCD member eligibility file who were missing information about insurance type. Because we compared care patterns in 2016 for those who received and did not receive care at FQHCs in 2015, we excluded enrollees with no outpatient visits in 2015 (*n* = 1,959), resulting in a final population of *N* = 32,330.

This study was approved by the Mass General Brigham Institutional Review Board for research related to secondary use of data, including a waiver of consent.

### Measures

We defined individuals as having received care at FQHCs in 2015 if they had at least one outpatient visit at a FQHC. Prior studies in the literature have used this definition to examine FQHC use as an independent variable, with no meaningful change in sensitivity analyses [24, 25]. Those with no FQHC visits but at least one outpatient visit in a non-FQHC setting were classified as not receiving care at FQHCs. To identify care occurring at FQHCs in the APCD, we linked billing and service National Provider Identifiers (NPI) on the claim to a set of NPIs manually identified as FQHCs based on their names, addresses, and taxonomy codes, as well as claims with FQHC-specific billing (site of service = 50 for professional claims and type of bill = 77 for facility claims) or FQHC-specific procedure codes (G0466-G0470, T1015 for Medicaid only).

Outpatient outcome measures included total number of outpatient visits at any setting and at FQHCs, mental health outpatient visits (visits with a primary mental health diagnosis or procedure codes G0469, G0470) at any setting and at FQHCs, and psychotropic medication fills in 2016. We examined the percentage of the cohort with at least one psychotropic medication fill. We additionally examined guideline recommended medication fills, including fills for antidepressants among those with diagnoses for depressive disorders only and antipsychotics for those with diagnoses of SSD. Acute care use outcomes included ED visits and hospitalizations in 2016. In secondary analyses, we classified visits or hospitalizations with a primary diagnosis of a mental health disorder (ICD-10 codes F01-F99) as psychiatric hospitalizations and ED visits. Inpatient claims with a service or billing NPI manually identified as a psychiatric hospital facility were also considered psychiatric hospitalizations. The APCD redacts claims for substance use disorders (SUD), so our analysis does not account for services utilized for co-occurring SUDs.

### Statistical analysis

We used multivariable linear regression models to assess associations between receipt of care at FQHCs in the prior year and the outcome measures. Given our large sample size, linear probability models were used for binary outcomes for ease of interpretation [26]. Models adjusted for individuals’ age, gender, whether they had any secondary commercial insurance, medical comorbidity as measured by the Charlson comorbidity index, and type of SMI diagnosis (depressive disorder, bipolar disorder, SSD), as well as ZIP code-level characteristics of race/ethnicity (% Hispanic, Non-Hispanic White, Non-Hispanic Black, Non-Hispanic Asian, Non-Hispanic Other) and low socioeconomic status (i.e., if >25% of population age 25+ did not graduate high school or if  >20% families had incomes <100% of the federal poverty level) [27, 28]. As a robustness check, we repeated the regression analysis including a fixed effect for county of residence as a proxy for unobservable neighborhood characteristics (Supplement File 2). All analyses were conducted using Stata 15 software.

## Results

### Study population characteristics

There were 8,887 (27.5%) adults in the study population (*N* = 32,330) who had at least one FQHC visit in 2015. Compared to those who did not receive care at FQHCs, those with FQHC use were more likely to be male and live in ZIP codes with lower socioeconomic status and a greater proportion of non-White residents, and less likely to have any months with secondary commercial insurance coverage (Table 1). Those with FQHC use were also more likely to have a bipolar or schizophrenia spectrum disorder and less likely to have a depressive disorder diagnosis.

**Table 1:** Characteristics of Medicaid adults with SMI in FQHC service areas who received outpatient care at FQHCs vs. other settings in 2015

Characteristics	Any care from FQHCs in 2015	Care only from non-FQHC settings in 2015	*p*-value
N	8,887	23,443	
Gender (%)			
Female	60.2	63.0	< 0.001
Male	39.8	37.0	
Age (%)			
18–25	10.5	10.5	< 0.001
26–40	29.5	26.3	
41–55	38.7	36.7	
56–64	21.4	26.4	
Insurance type (%)			
Any month with commercial insurance	6.0	8.7	< 0.001
ZIP code-level characteristics			
Low socioeconomic status (%)	39.4	29.4	< 0.001
ZIP code % Hispanic, mean ± SD	23.1 ± 20.4	19.5 ± 17.8	< 0.001
ZIP code % Non-Hispanic White, mean ± SD	52.8 ± 25.4	60.6 ± 24.0	< 0.001
ZIP code % Non-Hispanic Black, mean ± SD	14.1 ± 17.2	11.0 ± 14.2	< 0.001
ZIP code % Non-Hispanic Asian, mean ± SD	6.2 ± 7.2	5.3 ± 6.3	< 0.001
ZIP code % Non-Hispanic Other^a^, mean ± SD	3.8 ± 2.6	3.7 ± 2.5	0.001
Charlson Comorbidity Index, mean ± SD	1.19 ± 2.1	1.29 ± 2.1	< 0.001
2014–2015 mental health diagnoses (%)			
Depressive disorders	51.4	54.8	< 0.001
Bipolar disorder	30.4	28.9	
Schizophrenia spectrum disorders	18.2	16.2	

^a^Category “Non-Hispanic Other” included non-Hispanic individuals of American Indian / Alaska Native, Native Hawaiian/Other Pacific Islander races, and “Some other races”

### Outpatient care patterns for those receiving care at FQHCs vs. other settings

The mean total number of outpatient visits in 2016 was 19.0 for individuals who did and did not receive care at FQHCs in 2015; the mean number of FQHC visits in 2016 was 6.0 vs. 0.3, respectively (Table 2). Among those who received care at FQHCs in 2015, 67% had at least one visit with the FQHCs they visited most in 2015. Those who received care at FQHCs vs. other settings in the prior year were more likely to have at least one outpatient mental health visit (unadjusted percentage = 73.3% vs. 71.2%, adjusted difference = 2.6% points (pp), 95% CI: 1.5, 3.7). Among those with at least one mental health visit, the mean number of visits for those receiving care at FQHCs vs. other setting was slightly lower (10.5 vs. 11.2; adjusted difference=-0.48, 95% CI: -0.89, -0.07); among those with visits to FQHCs in the prior year, an average of 3.3 mental health visits occurred at FQHCs.

Of those receiving care at FQHCs in 2015, 73.2% vs. 69.0% of those receiving care in other settings filled at least one psychotropic medication in 2016 (adjusted difference = 4.7pp, 95% CI: 3.6, 5.8). Similarly, among those with depressive disorders, 61% of patients had at least one antidepressant fill vs. 58% of non-FQHC patients (adjusted difference 4.7pp, 95% CI: 3.0, 6.4); there was a similar difference in fills for antipsychotic medications among those with SSD (67.3% vs. 60.9%, adjusted difference 4.6pp, 95% CI: 1.8, 7.5).

**Table 2:** Differences in outpatient visits and psychotropic medication fills in 2016 for those who received care at FQHCs vs. other settings in 2015

Outcomes (2016)			Unadjusted		Adjusted		
	Any care from FQHCs in 2015	Care only from non-FQHC settings in 2015	Difference	*p*-value	Difference	95% CI	*p*-value
Number of outpatient visits, mean ± SD	19.0 ± 20.7	19.0 ± 20.0	0.010	0.97	0.79	0.30, 1.3	0.001
Number of visits at FQHCs, mean ± SD	6.0 ± 10.7	0.3 ± 2.0	5.7	< 0.001	5.6	5.5, 5.7	< 0.001
Percentage with 1+ mental health (MH) visit (%)	73.3	71.2	2.1	< 0.001	2.6	1.5, 3.7	< 0.001
Number of MH visits among those with 1+ MH visit, mean ± SD	10.5 ± 13.5	11.2 ± 14.8	-0.75	< 0.001	-0.48	-0.89, -0.07	0.023
Number of MH visits at FQHCs, mean ± SD	3.3 ± 6.3	0.2 ± 1.1	3.1	< 0.001	3.1	3.0, 3.2	< 0.001
Percentage who filled any psychotropic medication (%)	73.2	69.0	4.2	< 0.001	4.7	3.6, 5.8	< 0.001
Percentage with depressive disorders who filled antidepressants (%)	61.4	57.5	3.9	< 0.001	4.7	3.0, 6.4	< 0.001
Percentage with SSD who filled antipsychotics (%)	67.3	60.9	6.4	< 0.001	4.6	1.8, 7.5	0.001

### Acute care use for those receiving care at FQHCs vs. other settings

Those who received care at FQHCs vs. other settings were more likely to have ED visits (74.0% vs. 68.7%, adjusted difference = 5.1pp, 95% CI: 4.0, 6.3); findings were similar for medical and psychiatric ED visits (Table 3). Those with FQHC use were less likely to be hospitalized (27.8% vs. 31.9%, adjusted difference=-3.1pp, 95% CI: -4.1, -2.0); findings were again similar for medical hospitalizations, but there was no significant difference in the likelihood of having a psychiatric hospitalization.

**Table 3:** Differences in ED visits and hospitalizations in 2016 for those who received care at FQHCs vs. other settings in 2015

Outcomes (2016)			Unadjusted		Adjusted		
	Any care from FQHCs in 2015 (%)	Care only from non-FQHC settings in 2015 (%)	Difference	*p*-value	Difference	95% CI	*p*-value
Any ED visits	74.0	68.7	5.3	< 0.001	5.1	4.0, 6.3	< 0.001
Medical ED visits	60.2	58.2	2.0	0.001	2.5	1.3, 3.7	< 0.001
Psychiatric ED visits	25.0	18.2	6.8	< 0.001	5.8	4.8, 6.7	< 0.001
Any hospitalizations	27.8	31.9	-4.1	< 0.001	-3.1	-4.1, -2.0	< 0.001
Medical hospitalizations	20.1	23.8	-3.7	< 0.001	-2.6	-3.6, -1.6	< 0.001
Psychiatric hospitalizations	9.5	9.8	-0.26	0.48	-0.34	-1.0, 0.4	0.35

### Additional analyses

In sensitivity analysis that was limited to those with continuous Medicaid coverage in 2016 in addition to 2015, findings were consistent with the main analysis (Supplement file 1). In analyses that included county fixed effects, results were also similar (Supplement file 2). While point estimates were nearly identical to the main analysis, differences in the number of outpatient visits, number of mental health visits, percentage with SSD who filled antipsychotics for FQHC vs. non-FQHC patients were no longer significant at *p* < .05.

## Discussion

In a study population of Medicaid-insured adults with existing SMI diagnoses who were living in FQHC service areas, we found that over 1 in 4 had at least one outpatient visit to an FQHC. Although all individuals in the sample were living in areas with access to an FQHC, those with FQHC use tended to live in ZIP codes with greater socioeconomic disadvantage and a greater proportion of racial and ethnic minority residents. Although those with prior FQHC use had lower comorbidity scores, on average, they were more likely to have diagnoses of bipolar disorder or SSD and less likely to have depressive disorders alone, compared with those who received their outpatient care in other settings.

Individuals in this study population had an average of nearly 20 outpatient visits per year in 2016, including 10 mental health visits. Having prior FQHC use was associated with a modest increase in the probability of having an outpatient mental health visit and filling any psychotropic medication, including those consistent with clinical guidelines: i.e., antidepressants among those with depressive disorders and antipsychotics among those with SSD. Importantly, among those with prior FQHC use, we found that the majority of outpatient visits in 2016, including for mental health care, occurred outside of FQHCs. Nevertheless, individuals had an average of 6 outpatient visits per year to FQHCs, with half of those visits for a primary mental health concern.

Although there is scant prior evidence on care patterns for those with and without FQHC use in SMI populations, these findings align with observations about the role of FQHCs as primary medical care homes that connect patients with SMI to outside mental health services (e.g., community mental health centers, assertive community treatment teams), rather than acting as the main setting of mental health treatment [11, 29]. In a 2010 survey of FQHCs, among those providing mental health services, 25% of the mental health staff FTEs were licensed clinical social workers, 19% other licensed mental health providers, 25% other mental health staff, 17% unspecified staff providing substance use services, with psychiatrists and licensed clinical psychologist making up 7% each (FTE composition was similar in 2022) [3, 12]. Further implementing team-based approaches such as collaborative care will maximize the capacity of FQHC providers, care coordinators, and all other staff members to offer comprehensive evidence-based treatment to individuals with SMI.

Greater psychotropic medication use among those receiving care at FQHC is consistent with prior work demonstrating that Medicaid patients at FQHCs are less likely to have unmet need for prescription medications compared to those at other primary care settings [30]. On-site availability of pharmacy personnel has been cited as a potential facilitator of access to medication at FQHCs, as 77% of FQHCs had employed pharmacy staff per an analysis of the 2014 UDS [30]. FQHCs are also required to maintain accessibility of care in various domains, e.g., ensuring their hours of operation are responsive to patient needs, and there have also been regional quality improvement initiatives such as the Safety Net Medical Home Initiatives with the goal of advancing FQHC patient access to care [31]. 

This study found that FQHC patients were more likely to have ED visits but less likely to have medical hospitalizations. Medical hospitalizations have been associated with increased risk of mortality for those with SMI [32, 33]. Our findings differ from a previous study of Medicaid-covered individuals with SMI in North Carolina that found no difference in inpatient and ED utilization for FQHC patients vs. patients with other primary medical homes [10]. However, other studies of the general FQHC population have similarly found increases in ED visits and decreases in hospitalizations [34, 35]. Expansion of Medicaid coverage has been associated with increased ED utilization, [36–38] and one hypothesis for the phenomenon is that greater access to primary care may increase utilization across care settings, including the ED [36]. In contrast, we did not find a significant difference in psychiatric hospitalizations between those who received care at FQHCs vs. not, despite existing evidence that has demonstrated reductions in psychiatric hospitalizations associated with greater availability and intensity of community outpatient mental health services [39]. Continued research to identify effective approaches for reducing the need for acute inpatient psychiatric care is needed.

### Limitations

This is a non-randomized study. Our analysis limited the population to those with at least one outpatient visit and geographic access to FQHCs in the prior year, and we adjusted for a range of demographic and clinical measures; however, there could remain unmeasured differences between those with and without prior FQHC use. In addition, our outcome measures do not capture services utilized for co-occurring SUDs. FQHC capacity for SUD services is more limited compared to mental health services – in 2022, SUD services comprised 1.17% of all FQHC visits vs. mental health services being 7.87% of visits [12] – however, co-occurring substance use and SMIs are common, and future work should address utilization of SUD services among FQHC patients. In identifying mental health visits, we were unable to distinguish services rendered by primary care providers vs. mental health providers because provider specialty was not available in the dataset. While primary care clinicians provide critical mental health screening and treatment, it will be important to examine trends in mental health personnel capacity at FQHCs, especially for a population with SMIs. Generalizability may be limited as the study is specific to Massachusetts and the time period 2015–2016. Finally, we excluded individuals with no outpatient visits (about 6% of adult Medicaid enrollees with SMI) and did not have data on uninsured individuals.

## Conclusion

In a population of Medicaid enrollees with SMI, those who received care at FQHCs vs. other settings were more likely to have at least one outpatient mental health visit, fill psychotropic medications, and have emergency department visits, but were less likely to be hospitalized. FQHCs could serve as critical sources of care for patients with complex medical and psychiatric needs and facilitate care both within and outside of FQHCs for patients with SMI who often navigate distinct medical and mental health systems of care.

### Electronic supplementary material

Below is the link to the electronic supplementary material.

Supplementary Material 1

Supplementary Material 2

## Data Availability

Massachusetts All Payer Claim Database (APCD) Release 6.0: The data that support the findings of this study are available from the Center for Health Information and Analysis (CHIA) but restrictions apply to the availability of these data, which were used under license for the current study, and so are not publicly available. Data are however available from the authors upon reasonable request and with permission of CHIA. Uniform Data System (UDS) 2015: The datasets used and/or analyzed during the current study are available from the corresponding author on reasonable request. American Community Survey 5-year Data 2011-15: The datasets generated and/or analyzed during the current study are available in the American Community Survey repository, https://www2.census.gov/programs-surveys/acs/. Dataset of NPIs associated with FQHCs in MA: The datasets used and/or analyzed during the current study are available from the corresponding author on reasonable request. Dataset of MA ZIP codes in empirically designated FQHC service areas: The datasets used and/or analyzed during the current study are available from the corresponding author on reasonable request.

## Abbreviations

FQHC: Federally qualified health center
SMI: Serious mental illness
SSD: Schizophrenia spectrum disorder
ED: Emergency department
APCD: All Payer Claim Database
UDS: Uniform Data System
HRSA: Health Resources and Services Administration
NPI: National Provider Identifier
ICD: International Classification of Diseases
SUD: Substance use disorder

## References

[1] De Hert M, Dekker JM, Wood D, Kahl KG, Holt RIG, Möller HJ (2009). Cardiovascular disease and diabetes in people with severe mental illness position statement from the European Psychiatric Association (EPA), supported by the European Association for the Study of Diabetes (EASD) and the European Society of Cardiology (ESC). Eur Psychiatry.

[2] Surtees PG, Wainwright NW, Luben RN, Wareham NJ, Bingham SA, Khaw KT (2008). Depression and ischemic heart disease mortality: evidence from the EPIC-Norfolk United Kingdom prospective cohort study. Am J Psychiatry.

[3] Brolin M, Quinn A, Sirkin JT, Horgan CM, Parks J, Easterday J, et al. Financing of Behavioral Health Services within federally qualified Health centers. Substance Abuse and Mental Health Services Administration; 2012.

[4] Coombs NC, Meriwether WE, Caringi J, Newcomer SR (2021). Barriers to healthcare access among U.S. adults with mental health challenges: a population-based study. SSM Popul Health.

[5] Dedania R, Gonzales G (2019). Disparities in Access to Health Care among US-Born and Foreign-born US adults by Mental Health Status, 2013–2016. Am J Public Health.

[6] Ostrow L, Manderscheid R, Mojtabai R (2014). Stigma and difficulty accessing medical care in a sample of adults with serious mental illness. J Health Care Poor Underserved.

[7] Strine TW, Zack M, Dhingra S, Druss B, Simoes E (2011). Uninsurance among nonelderly adults with and without frequent mental and physical distress in the United States. Psychiatr Serv.

[8] SAMHSA. Serious Mental Illness Among Adults Below the Poverty Line. https://www.samhsa.gov/data/sites/default/files/report_2720/Spotlight-2720.html. Accessed 24 Jan 2024.

[9] Saunders H, Rudowitz R, Demographics. Jan and Health Insurance Coverage of Nonelderly Adults With Mental Illness and Substance Use Disorders in 2020. https://www.kff.org/mental-health/issue-brief/demographics-and-health-insurance-coverage-of-nonelderly-adults-with-mental-illness-and-substance-use-disorders-in-2020/. Accessed 24 2024.

[10] Kilany M, Wells R, Morrissey JP, Domino ME (2021). Are there Performance Advantages Favoring Federally Qualified Health Centers in Medical Home Care for persons with severe Mental illness?. Adm Policy Ment Health.

[11] Kristopher E, Kaliebe MD (2016). The Future of Psychiatric Collaboration in Federally Qualified Health Centers. Psychiatric Serv.

[12] UDS Data Five-Year. Summary [Internet]. Health Resources & Services Administration. https://data.hrsa.gov/tools/data-reporting/program-data/national. Accessed 24 Jan 2024.

[13] Lewis C, Coleman A, Abrams MK, Doty MM. The role of Medicaid Expansion in Care Delivery at Community Health Centers. The Commonwealth Fund; 2019.

[14] Health Center Service Expansion. https://www.nachc.org/wp-content/uploads/2023/07/Service-Expansion-Issue-Brief_2023_print-ready_final.pdf. Accessed 24 Jan 2024.

[15] Bruckner TA, Singh P, Snowden LR, Yoon J, Chakravarthy B (2019). Rapid Growth of Mental Health Services at Community Health Centers. Adm Policy Ment Health.

[16] Neuhausen K, Grumbach K, Bazemore A, Phillips RL (2012). Integrating community health centers into organized delivery systems can improve access to subspecialty care. Health Aff (Millwood).

[17] Goldstein EV. Integrating Mental and Physical Health Care for Low-Income Americans: Assessing a Federal Program’s Initial Impact on Access and Cost. Healthc (Basel). 2017;5(3). 10.3390/healthcare5030032 PMC5618160 28704970

[18] Jones EB, Ku L (2015). Sharing a playbook: Integrated Care in Community Health Centers in the United States. Am J Public Health.

[19] Whitaker RG, Kilany M, Wells R, Domino ME (2021). Are Certain Health Centers Better Patient-Centered Medical Homes for People with severe Mental illness?. Psychiatr Q.

[20] Brown ER, Davidson PL, Yu H, Wyn R, Andersen RM, Becerra L (2004). Effects of Community factors on Access to Ambulatory Care for lower-income adults in large Urban communities. INQUIRY: J Health Care Organ Provis Financing.

[21] Behr CL, Hull P, Hsu J, Newhouse JP, Fung V (2022). Geographic access to federally qualified health centers before and after the affordable care act. BMC Health Serv Res.

[22] Busch AB, Yoon F, Barry CL, Azzone V, Normand SL, Goldman HH (2013). The effects of mental health parity on spending and utilization for bipolar, major depression, and adjustment disorders. Am J Psychiatry.

[23] Fung V, Price M, Busch AB, Landrum MB, Fireman B, Nierenberg A (2013). Adverse clinical events among medicare beneficiaries using antipsychotic drugs: linking health insurance benefits and clinical needs. Med Care.

[24] Wright B, Potter AJ, Trivedi AN (2017). Use of Federally Qualified Health Centers and potentially preventable hospital utilization among older Medicare-Medicaid enrollees. J Ambul Care Manage.

[25] Potter AJ, Trivedi AN, Wright B (2017). Younger dual-eligibles who use federally qualified Health centers have more preventable Emergency Department visits, but some have fewer hospitalizations. J Prim Care Community Health.

[26] Long JS. Regression models for categorical and limited dependent variables. SSAGE Publications, Inc; 1997. p. 328.

[27] Krieger N, Waterman PD, Chen JT (2020). COVID-19 and overall mortality inequities in the Surge in Death Rates by Zip Code characteristics: Massachusetts, January 1 to May 19, 2020. Am J Public Health.

[28] Subramanian SV, Chen JT, Rehkopf DH, Waterman PD, Krieger N (2006). Comparing individual- and area-based socioeconomic measures for the surveillance of health disparities: a multilevel analysis of Massachusetts births, 1989–1991. Am J Epidemiol.

[29] Henwood BF, Siantz E, Hrouda DR, Innes-Gomberg D, Gilmer TP (2018). Integrated Primary Care in Assertive Community Treatment. Psychiatr Serv.

[30] Pourat N, Chen X, Lee C, Zhou W, Daniel M, Hoang H (2019). HRSA-funded Health centers are an important source of Care and reduce unmet needs in primary care services. Med Care.

[31] Adashi EY, Geiger HJ, Fine MD (2010). Health Care Reform and Primary Care — the growing importance of the Community Health Center. N Engl J Med.

[32] Qin P, Webb R, Kapur N, Sørensen HT (2013). Hospitalization for physical illness and risk of subsequent suicide: a population study. J Intern Med.

[33] Ribe AR, Vestergaard M, Katon W, Charles M, Benros ME, Vanderlip E (2015). Thirty-day mortality after infection among persons with severe Mental illness: a Population-based Cohort Study in Denmark. Am J Psychiatry.

[34] Knitter AC, Murugesan M, Saulsberry L, Wan W, Nocon RS, Huang ES (2022). Quality of care for US adults with Medicaid Insurance and Type 2 diabetes in federally qualified Health centers compared with other primary care settings. Med Care.

[35] Laiteerapong N, Kirby J, Gao Y, Yu TC, Sharma R, Nocon R (2014). Health care utilization and receipt of preventive care for patients seen at federally funded health centers compared to other sites of primary care. Health Serv Res.

[36] Finkelstein AN, Taubman SL, Allen HL, Wright BJ, Baicker K (2016). Effect of Medicaid Coverage on ED use — further evidence from Oregon’s experiment. N Engl J Med.

[37] Zhao F, Nianogo RA (2022). Medicaid Expansion’s impact on Emergency Department Use by State and Payer. Value Health.

[38] Sommers BD, Simon K (2017). Health Insurance and Emergency Department Use — A Complex Relationship. N Engl J Med.

[39] Wanchek TN, McGarvey EL, Leon-Verdin M, Bonnie RJ (2011). The effect of community mental health services on hospitalization rates in Virginia. Psychiatr Serv.

