# Supplementary material for: Mental health care use and quality among Medicaid adults with serious mental illness receiving care at Federally Qualified Health Centers vs. other settings
Source: BMC Health Serv Res. 2024 Jul 17;24:825. doi: 10.1186/s12913-024-11308-1 (PMC11256553; doi:10.1186/s12913-024-11308-1)
Supplement: Supplementary file 1 — Supplementary Material 1 [file 12913_2024_11308_MOESM1_ESM.docx]

Supplementary file 1 Table 1. Sensitivity analysis excluding n = 3263 individuals without continuous Medicaid enrollment in 2016: Characteristics of Medicaid adults with SMI in FQHC service areas who received outpatient care at FQHCs vs. other settings in 2015

| Characteristics | Any care from FQHCs in 2015 | Care only from non-FQHC settings in 2015 | p-value |
| --- | --- | --- | --- |
| N | 8040 | 21027 |  |
| Gender (%) |  |  |  |
| Female | 61.2 | 63.6 | <0.001 |
| Male | 38.8 | 36.4 |  |
| Age (%) |  |  |  |
| 18-25 | 10.3 | 10.5 | <0.001 |
| 26-40 | 29.2 | 25.8 |  |
| 41-55 | 38.9 | 37.1 |  |
| 56-64 | 21.6 | 26.6 |  |
| Insurance type (%) |  |  |  |
| Any month with commercial insurance | 5.8 | 8.0 | <0.001 |
| ZIP code-level characteristics |  |  |  |
| Low socioeconomic status (%) | 40.0 | 29.8 | <0.001 |
| ZIP code % Hispanic, mean ± SD | 23.3 ± 20.4 | 19.6 ± 17.8 | <0.001 |
| ZIP code % Non-Hispanic White, mean ± SD | 52.5 ± 25.4 | 60.4 ± 24.1 | <0.001 |
| ZIP code % Non-Hispanic Black, mean ± SD | 14.2 ± 17.3 | 11.0 ± 14.3 | <0.001 |
| ZIP code % Non-Hispanic Asian, mean ± SD | 6.2 ± 7.2 | 5.3 ± 6.3 | <0.001 |
| ZIP code % Non-Hispanic Other^a^, mean ± SD | 3.8 ± 2.6 | 3.7 ± 2.5 | 0.003 |
| Charlson Comorbidity Index, mean ± SD | 1.16 ± 2.0 | 1.27 ± 2.1 | <0.001 |
| 2014-2015 mental health diagnoses (%) |  |  |  |
| Depressive disorders | 50.9 | 54.2 | <0.001 |
| Bipolar disorder | 30.4 | 29.0 |  |
| Schizophrenia spectrum disorders | 18.6 | 16.8 |  |

^a^Category “Non-Hispanic Other” included non-Hispanic individuals of American Indian / Alaska Native, Native Hawaiian/Other Pacific Islander races, and “Some other races”.

Supplementary file 1 Table 2. Sensitivity analysis excluding n = 3263 individuals without continuous Medicaid enrollment in 2016: Differences in outpatient visits and psychotropic medication fills in 2016 for those who received care at FQHCs vs. other settings in 2015

| Outcomes (2016) |  | | Unadjusted | | Adjusted | | |
| --- | --- | --- | --- | --- | --- | --- | --- |
|  | Any care from FQHCs in 2015 | Care only from non-FQHC settings in 2015 | Difference | p-value | Difference | 95% CI | p-value |
| Number of outpatient visits, mean ± SD | 20.1 ± 21.1 | 20.2 ± 20.4 | -0.079 | 0.77 | 0.80 | 0.28,1.32 | 0.003 |
| Number of visits at FQHCs | 6.4 ± 11.1 | 0.4 ± 2.1 | 6.0 | <0.001 | 5.9 | 5.8,6.1 | <0.001 |
| Percentage with 1+ mental health (MH) visit (%) | 76.1 | 73.8 | 2.3 | <0.001 | 2.9 | 1.8,4.1 | <0.001 |
| Number of MH visits among those with 1+ MH visit, mean ± SD | 10.7 ± 13.8 | 11.6 ± 15.1 | -0.88 | <0.001 | -0.58 | -1.0,-0.14 | 0.009 |
| Number of MH visits at FQHCs | 3.3 ± 6.4 | 0.2 ± 1.1 | 3.1 | <0.001 | 3.2 | 3.0,3.3 | <0.001 |
| Percentage who filled any psychotropic medication (%) | 74.8 | 70.3 | 4.5 | <0.001 | 4.9 | 3.7,6.0 | <0.001 |
| Percentage with depressive disorders who filled antidepressants (%) | 62.8 | 58.8 | 4.0 | <0.001 | 4.6 | 2.8,6.3 | <0.001 |
| Percentage with SSD who filled antipsychotics (%) | 69.2 | 62.4 | 6.8 | <0.001 | 5.1 | 2.2,8.0 | 0.001 |

Supplementary file 1 Table 3. Sensitivity analysis excluding n = 3263 individuals without continuous Medicaid enrollment in 2016: Differences in ED visits and hospitalizations in 2016 for those who received care at FQHCs vs. other settings in 2015

| Outcomes (2016) |  | | Unadjusted | | Adjusted | | |
| --- | --- | --- | --- | --- | --- | --- | --- |
|  | Any care from FQHCs in 2015 (%) | Care only from non-FQHC settings in 2015 (%) | Difference | p-value | Difference | 95% CI | p-value |
| Any ED visits | 75.5 | 70.3 | 5.2 | <0.001 | 5.1 | 3.9,6.3 | <0.001 |
| Medical ED visits | 61.1 | 59.5 | 1.6 | 0.01 | 2.3 | 1.0, 3.5 | <0.001 |
| Psychiatric ED visits | 25.8 | 18.9 | 6.9 | <0.001 | 6.0 | 5.0,7.0 | <0.001 |
| Any hospitalizations | 28.2 | 32.7 | -4.5 | <0.001 | -3.4 | -4.6,-2.2 | <0.001 |
| Medical hospitalizations | 20.2 | 24.3 | -4.1 | <0.001 | -2.8 | -3.9,-1.8 | <0.001 |
| Psychiatric hospitalizations | 9.8 | 10.2 | -0.4 | 0.26 | -0.47 | -1.2,0.3 | 0.22 |
