# Supplementary material for: Mental health care use and quality among Medicaid adults with serious mental illness receiving care at Federally Qualified Health Centers vs. other settings
Source: BMC Health Serv Res. 2024 Jul 17;24:825. doi: 10.1186/s12913-024-11308-1 (PMC11256553; doi:10.1186/s12913-024-11308-1)
Supplement: Supplementary file 2 — Supplementary Material 2 [file 12913_2024_11308_MOESM2_ESM.docx]

| Outcomes (2016) |  | | Unadjusted | | Adjusted | | |
| --- | --- | --- | --- | --- | --- | --- | --- |
|  | Any care from FQHCs in 2015 | Care only from non-FQHC settings in 2015 | Difference | p-value | Difference | 95% CI | p-value |
| Number of outpatient visits, mean ± SD | 19.0 ± 20.7 | 19.0 ± 20.0 | 0.010 | 0.97 | 0.84 | -0.8,2.5 | 0.28 |
| Number of visits at FQHCs | 6.0 ± 10.7 | 0.3 ± 2.0 | 5.7 | <0.001 | 5.5 | 4.5,6.5 | <0.001 |
| Percentage with 1+ mental health (MH) visit (%) | 73.3 | 71.2 | 2.1 | <0.001 | 2.7 | 0.5,4.8 | 0.019 |
| Number of MH visits among those with 1+ MH visit, mean ± SD | 10.5 ± 13.5 | 11.2 ± 14.8 | -0.75 | <0.001 | -0.44 | -1.6,0.7 | 0.43 |
| Number of MH visits at FQHCs | 3.3 ± 6.3 | 0.2 ± 1.1 | 3.1 | <0.001 | 3.0 | 2.0, 4.0 | <0.001 |
| Percentage who filled any psychotropic medication (%) | 73.2 | 69.0 | 4.2 | <0.001 | 4.4 | 2.9,5.8 | <0.001 |
| Percentage with depressive disorders who filled antidepressants (%) | 61.4 | 57.5 | 3.9 | <0.001 | 4.7 | 3.0, 6.4 | <0.001 |
| Percentage with SSD who filled antipsychotics (%) | 67.3 | 60.9 | 6.4 | <0.001 | 4.6 | -0.6, 7.8 | 0.087 |

Supplementary file 2 Table 1. Regression analysis including county-level fixed effects with robust standard errors: Differences in outpatient visits and psychotropic medication fills in 2016 for those who received care at FQHCs vs. other settings in 2015

Supplementary file 2 Table 2. Regression analysis including county-level fixed effects with robust standard errors: Differences in ED visits and hospitalizations in 2016 for those who received care at FQHCs vs. other settings in 2015

| Outcomes (2016) |  | | Unadjusted | | Adjusted | | |
| --- | --- | --- | --- | --- | --- | --- | --- |
|  | Any care from FQHCs in 2015 (%) | Care only from non-FQHC settings in 2015(%) | Difference | p-value | Difference | 95% CI | p-value |
| Any ED visits | 74.0 | 68.7 | 5.3 | <0.001 | 5.1 | 4.1, 6.1 | <0.001 |
| Medical ED visits | 60.2 | 58.2 | 2.0 | 0.001 | 2.6 | 1.4, 3.7 | <0.001 |
| Psychiatric ED visits | 25.0 | 18.2 | 6.8 | <0.001 | 5.6 | 3.9, 7.3 | <0.001 |
| Any hospitalizations | 27.8 | 31.9 | -4.1 | <0.001 | -3.1 | -4.7, -1.5 | 0.001 |
| Medical hospitalizations | 20.1 | 23.8 | -3.7 | <0.001 | -2.7 | -4.2, -1.3 | 0.001 |
| Psychiatric hospitalizations | 9.5 | 9.8 | -0.26 | 0.48 | -0.19 | -1.6, 1.2 | 0.77 |
